# A Case of Fistula in Ano, with Remarks

**Published:** 1879-06

**Authors:** 


### Selections.

### A CASE OF FISTULA IN ANO, WITH REMARKS.

Mr. W. L. C. was brought to me in November last, by my friend, Dr. E. O. Reid, of Milburn, for an operation for fistula in ano. The following is the history of the patient so far as could be learned, together with a description of his condition upon presentation to me.. Age 38, married, occupation a farmer. Twenty years ago he had fallen from a wagon and struck the left buttock against the end of a log. About five years after this accident an abscess formed near the point of injury. This opened of itself and left a sinus, which continued for twelve years as a simple fistula, without causing much pain or uneasiness. At this time, however, the discharge of pus began gradually to increase, and more pain than usual set in. He consulted a number of physicians from this on, until the time that I saw him. They usually prescribed some ointment, or burnt the sinus with caustics, all of course to no avail. At the end of four more years, making it three years prior to the time that I saw him, he began to lose flesh. The discharge of pus greatly increased; the pain was intense, and he was unable to attend to any duties upon the farm. Poulticing was resorted to, to quiet the pain. This increased the flow of pus, hence debility rapidly increased. After the lapse of another two years he was confined to his bed, and was unable to rest in any position, save on his stomach. This condition continued up to the time that he was brought for operation, November 25, 1878. At this time he presented the following symptoms, etc.: Great emaciation, but little appetite, unable to stand from excessive debility; profuse night sweats, and a discharge of pus, fully a half pint per day; feet and legs œdematous; pulse 120 and very feeble; temperature above normal; entire left buttock much enlarged and œdematous; left leg very stiff. Upon examination for fistula, there was found at a point corresponding to the triangular space, just in front of the transversus perinei muscle of the left side, a large opening, from which pus rapidly escaped when pressure was made upon the left buttock. On the introduction of a probe it took a direction back through the ischio rectal fossa, and again to the front, rather superficially over the perineal muscles. The probe could not be passed into the bowel from the sinus, though upon examination for internal openings, a large one was found dorsally situated, through which water could be forced by syringe introduced at the external opening bordering on the perineum. Another external opening existed dorsally, a little to the right of the coccyx. This had no communication with the bowel.

I confess that to operate in this case was risky, for several reasons: First, should an operation be performed at all, was it not necessary to "build up" the patient before attempting it? Or, secondly, should we decline to operate, accepting the teachings of many that "fistula is an issue established by nature as a remedy for pulmonary disease," and that to stop the discharge would be to send the patient to a premature death; or, third, that to stop the discharge pulmonary disease would, as some believe, be established. I beg pardon for remarking that this latter belief is held only by the laity. Were we to consult authorities upon the subject of the propriety of an operation under the circumstances narrated, we would at once throw away the knife, for it is the universal teaching that the patient's physical condition at the time of the operation must be good, else the risk of failure is great. Upon general rules of practice I admit the plausibility of this reasoning. But here we have a man who is on the road to death from an excessive discharge of pus. He is beyond that stage when to wait and build up would profit him anything. The discharge would over-balance in loss what could be gained by supportive or other treatment. In other words, to dally would be to let the patient die. Under this train of reasoning I proceeded to operate, and I am glad to say that the result bears me out in that conclusion. Chloroform was given by Dr. E. O. Reid, and my friend Dr. G. J. Cook lent me his valuable assistance in the operation. Introducing a grooved director through the dorsal opening, it was carried through the mucous membrane into the bowel and brought out at the anus; an incision was then made through the mucous sphincter with a bistoury. The director was then introduced into the external opening in the perineum and pushed forward toward the scrotum, making a superficial cut about two inches in length, then pushing the director backwards, it passed into the ischio-rectal fossa. It ran back about six inches. A free incision was then made dividing all the structures upon the director. Searching at the bottom of the wound, it was discovered that from about its middle another sinus ran towards the hip-joint. The knife was carried as freely into the parts as could be done, passing in close proximity to the internal pudic artery. Another deep-seated sinus, running into the bowel, was observed, but was not laid open at the operation, because of the serious results apprehended from cutting twice through the sphincter-ani muscle. The wounds made were large enough to admit the hand, and were dressed with the solution persulph. iron, and cotton wool placed firmly at the bottom. The patient was then allowed to come from under the influence of chloroform, which he did without any untoward symptom, save nausea. The next day he was put upon ext. malt and iron, with a due allowance of port wine. Was freely fed with nutritious food. For several days his pulse and temperature were lower; appetite slightly increased. Upon examination of the wound pus was found freely escaping. This continued for three weeks, when it became evident that it was due to other causes, and that the wounds would not heal inasmuch as the pus passed freely over their surfaces. The cut into the bowel from dorsal region having nearly healed, it was deemed expedient to perform a second operation, and lay open, if possible, the remaining sinuses. Assisted by my friends Drs. G. J. Cook, and M. F. Comes, I proceeded to operate. The patient having been put under the influence of chloroform by Dr. Comes, I explored the bottom of the wound, and passing the director down in to a sinus it was carried into the bowel; all the tissues then on the director were divided. Searching at the bottom of this cut, what was my surprise to find two large additional sinuses passing away back beneath the glutæi muscles and forming a sack at the bottom. It being impossible to cut deeper, I was content to freely scarify the walls of each sinus, which were hard and calloused. The patient lost considerable blood at this operation, though no tying of vessels was necessary. The small vessels around the cartilage-like tissue were in a condition of "canalesis" and a good deal of oozing took place. This ceased after a very free application of the persulph iron Cotton wool was gently packed to the bottom of all the wounds and a T bandage applied. Nausea again supervened, but the patient next morning expressed himself as feeling much easier.

The supportive treatment was continued, and after three days the dressings were removed. The amount of pus gradually diminished from this day. The pulse fell; temperature approached normal; appetite came up, and night sweats ceased. The wounds were freely syringed each day with carbolized water, and dressed with cotton wool. Granulations began to set up at the bottom of each wound; a gradual filling up took place. The patient's general health continued to improve. After a week he could sleep on his back with comfort (which he had not done before for four years). No pus escaped save from cut surfaces. Œdema of feet, legs and buttock had disappeared; could draw the limb fully up with the other one. On February 28, just three months from the time that he came under treatment, I discharged him, cured. On that day he walked to the boat, a distance of seven squares, and carried a valise. He had gained more than twenty pounds in flesh. After a month at home he writes me that his general health is excellent, that he sleeps soundly, that there is no pain whatever in the part, and that he goes to town, a distance of three miles, twice a week. Before closing the account of this case I beg to call the attention of those who may read it to four special points, without comment, viz:

1. The length of time the disease had existed.

2. The physical condition of the patient when operated on.
3. The extensive cutting that was done.
4. The complete recovery of the patient.—*Med. Herald.*

